# Classical Scrapie Did Not Re-occur in Goats After Cleaning and Disinfection of the Farm Premises

**DOI:** 10.3389/fvets.2020.00585

**Published:** 2020-09-02

**Authors:** Timm Konold, Sonja Libbey, Brenda Rajanayagam, Louise Fothergill, John Spiropoulos, Beatriz Vidaña, Pablo Alarcon

**Affiliations:** ^1^Pathology Department, Animal and Plant Health Agency Weybridge, Addlestone, United Kingdom; ^2^Animal and Plant Health England Field Delivery, Dorchester, United Kingdom; ^3^Department of Epidemiological Sciences, Animal and Plant Health Agency Weybridge, Addlestone, United Kingdom; ^4^Genotyping Unit, Animal and Plant Health Agency Weybridge, Addlestone, United Kingdom; ^5^Faculty of Health Sciences, Bristol Veterinary School, University of Bristol, Langford, United Kingdom; ^6^Department of Pathobiology and Population Sciences, Royal Veterinary College, University of London, Hatfield, United Kingdom

**Keywords:** transmissible spongiform encephalopathy, scrapie, goats, disinfection, immunohistochemical examination

## Abstract

After an outbreak of classical scrapie in a dairy goat herd with over 1,800 goats, all goats in the herd were culled in 2008, cleaning and disinfection of the premises was implemented, and restocking with goats took place ~4 months after depopulation. Ten years later the new herd population is over 3,000 goats. This study was carried out to determine whether the measures were effective to prevent re-occurrence of scrapie to the 1% prevalence level seen when scrapie was first detected on this farm. A total of 280 goats with a minimum age of 18 months, which were predominantly at the end of their productive life, were euthanized, and brain and retropharyngeal lymph node examined by immunohistochemistry for disease-associated prion protein. Genotyping was done in all euthanized goats and live male goats used or intended for breeding to determine prion protein gene polymorphisms associated with resistance to classical scrapie. None of the goats presented with disease-associated prion protein in the examined tissues, and 34 (12.2%) carried the K_222_ allele associated with resistance. This allele was also found in four breeding male goats. The study results suggested that classical scrapie was not re-introduced on this goat farm through mass restocking or inadequate cleaning and disinfection procedures. Further scrapie surveillance of goats on this farm is desirable to confirm absence of disease. Breeding with male goats carrying the K_222_ allele should be encouraged to increase the scrapie-resistant population.

## Introduction

Classical scrapie is a naturally occurring transmissible spongiform encephalopathy in sheep and goats. The infectious agent, the prion, is extremely resistant to disinfection and can persist in the environment for many years, which increases the risk of re-infection if susceptible animals are reintroduced to a contaminated farm or building. It has been shown that sheep became infected after being housed in a contaminated barn 16 years after its last use to house sheep (1). In the UK, pressure washing and subsequent disinfection with 2% sodium hypochlorite for an hour, which has been shown to be effective against scrapie strain 263K in hamsters (2), is the standard practice for disinfection of scrapie-contaminated premises. Yet, this measure, and even replacement or re-galvanization of metalwork and re-painting did not prevent re-occurrence of scrapie in an experimental sheep farm with a high incidence of naturally transmitted scrapie (3). High-level cleaning followed by exposure of surfaces with 2% sodium hypochlorite for an hour, repeated three times, was equally not effective to prevent re-infection of sheep in a scrapie-contaminated barn (4). The common feature of these studies was the reintroduction of sheep with highly susceptible prion protein gene (*PRNP*) genotypes in potentially scrapie-contaminated areas, which may be the worst-case scenario and may not always reflect the natural situation where sheep of different *PRNP* genotypes may be present. It has been known for some time that susceptibility to classical scrapie is influenced by the *PRNP* genotype and polymorphisms at codons 136, 154, and 171 in sheep: VRQ-encoding genotypes have the greatest scrapie risk, whereas ARR/ARR sheep have the lowest risk (5). Indeed, European legislation on the eradication of transmissible spongiform encephalopathies (TSEs) in small ruminants provides several options when dealing with an outbreak of classical scrapie in sheep flocks, which includes derogations to the destruction of sheep from infected flocks if sheep carry resistant genotypes, and different movement restrictions and rules on the introduction of new animals in holdings depending on genotypes (6). The European legislation on TSE eradication (7) has recently been amended to include a genotype-based approach for disease eradication also for goats, based on the existence of resistant genotypes in this species as well: at codons 146 (D or S instead of N) and 222 (K instead of Q) (8, 9). Eradication efforts may have however been hampered by the low frequency of these resistant genotypes (10–12). The legislation currently allows restocking of farms with goats that have been depopulated following an outbreak of classical scrapie provided that a cleaning and disinfection (C&D) of all animal housing on the premises has been carried out following destocking (7). Whilst there is a risk of subsequently re-introducing the disease through the introduction of replacement stock (13), nothing is known about the risk of re-infection of goats introduced to potentially contaminated farms. To fill this gap, a study was carried out to monitor re-occurrence of scrapie after depopulation of a goat farm following an outbreak of classical scrapie (14), which was subsequently restocked after standard C&D but not subjected to scrapie monitoring by active surveillance for 8 years, although the farmer was required to remain vigilant to report clinical suspects because of the farm's history. Based on the situation in sheep, it was hypothesized that persistence of the infectious agent would lead to re-introduction of classical scrapie. The aim of the study was therefore to determine freedom of infection to at least a 1% prevalence level in this goat herd.

## Method

### Herd History

Historical data on this herd, which had the first case of scrapie confirmed in 2005, was published previously (14). Briefly, it was an intensively managed dairy herd that was permanently housed indoors. At the time of whole herd cull in March 2008, 1,820 goats of three breeds (Anglo-Nubian, Saanen, and Toggenburg) were present. Annual scrapie prevalence in the herd was 0.5, 2.4, and 5% in the years 2005-6, 2006-7, and 2007-8 respectively. This rise in the infection rate contributed to the decision to cull the whole herd according to legislative requirement at that time.

After depopulation, C&D was applied, and the farm was restocked with new goats ~4 months after depopulation. C&D involved removal of all loose dirt from the walls. This was followed by pressure washing of walls and ceiling and disinfection with sodium hypochlorite although there were no records on the concentration and exposure time used. Top soil from the building's floor was removed up to the bottom chalk layer, which was then covered with lime. All wooden timber was replaced with new wood or steel structures, and new milking equipment was also installed. Manure, including the manure that was collected during the scrapie outbreak, was composted in a pit that was emptied at least annually and used as fertilizer for pasture land, which was used to produce grass and maize and fed back to the goats. The new goat population would have been exposed to feedstuffs fertilized with composted manure from scrapie-affected goats.

The new goat population was housed in the old building, although three new buildings were constructed seven years after depopulation, one to house dairy goats, one occupied by the milking parlor, and one separate building to rear kids, which was located 200 acres away from the main farm site.

The farm was repopulated with the purchase of 1,479 dairy goats from one farm with high health status, which had participated in the scrapie surveillance program prior to purchase, with 114 goats tested as fallen stock with negative results between 2005 and 2007. In addition, the owner purchased 13 male goats from 5 different farms between 2008 and 2013, of which four had some (1–23) goats tested in the scrapie surveillance program, with negative results. None of the farms had reported any scrapie suspect cases. Two goat farms and 15 sheep farms were located within a 3 km radius; and 36 goat farms and 123 sheep farms were detected within a 10 km radius, none of which had any scrapie cases detected through active or passive surveillance. Active scrapie monitoring of goats that died on the farm up to 2 years after restocking did not detect any scrapie cases; 131 were tested in 2009 and 129 tested in 2010.

At the time of the study, the farm had 3,100 goats, none of which had been genotyped. The majority were Saanen (42%) pure or crossbreeds, followed by Anglo-Nubian (28%), Toggenburg (18%) and British Alpine goats (12%). Of these, 48% were 18 months or younger, 24% where between 18 months and 3 years old, 17% were between 3 and 6 years old, and 11% were 6 years or older.

### Postmortem Examination

As confirmatory tests were superior in their diagnostic sensitivity compared to the rapid TSE test currently used in the UK (14, 15), only immunohistochemistry (IHC) to detect scrapie-associated prion protein (PrP^Sc^) was used. The following tissues were collected from euthanized goats for examination by IHC: retropharyngeal lymph node (RPLN), obex and cerebellum. The lymph node from one side, half of the obex cut sagittally and the whole cerebellum were fixed in 10% buffered formalin. Tissue processing and immunolabeling with monoclonal antibody R145 (APHA Weybridge, New Haw, UK) for IHC was carried out as described previously (15).

### Genotyping

A piece of ear was submitted fresh from each goat and DNA extracted using the Qiagen Dneasy Blood & Tissue kit (Qiagen Ltd., Manchester, UK) according to the manufacturer's instructions. The full open reading frame of the caprine *PRNP* was determined using the same equipment, reagents, primers and protocol as published previously (16) and to check especially for variant haplotypes associated with partial resistance to scrapie, such as M_142_, R_143_, S_146_ or D_146_, H_154_, Q_211_ and K_222_ (9). Following the genotyping results, which indicated some resistance to classical scrapie in the tested population, all males used or intended for breeding were subsequently blood sampled for genotyping to determine *PRNP* polymorphisms in particular at codon 222.

### Sampling Population

For the sample size calculations the following was considered:

Only animals over one year of age were considered, which was estimated to be 71.8% of the herd (*n* = 2226). The target herd sensitivity was 95%, meaning that if an animal was scrapie positive on farm, there was 95% confidence that it would be detected.

For test sensitivity, data were used from a separate study in goats (17) that was directly comparable because of identical use of tests [rapid test Bio-Rad ELISA (TeSeE ELISA, Bio-Rad Laboratories, Watford, UK) and IHC] and examined tissues (brain and RPLN). In that study, examination of brain and RPLN by IHC had a sensitivity of 97.4%, with 114 of 117 goats with scrapie confirmed by this method whilst Bio-Rad ELISA alone detected 61 (52.1%) of cases.

The design prevalence was estimated to be 1%, which was based on the initial incidence rate in 2005 (0.5%) using only the rapid TSE test (Bio-Rad ELISA) with ~50% sensitivity and therefore a double prevalence (1%) would have been expected.

The sample size was then calculated based on published recommendations (18) using the online tool for demonstration of freedom (detection of disease) in a finite population (https://epitools.ausvet.com.au/freedomfinitepop) with the parameters listed above (expected prevalence of 1%). This resulted in a sample size of 280 goats to test.

## Results

The final results with animal, genotype and test data are provided as Supplementary File.

All animals tested negative for scrapie by IHC on brain and lymphoid tissue. Suitable RPLN tissue was not available from four (1.4%) goats. Another seven (2.5%) goats had brainstem tissue collected, which did not include the target areas [parasympathetic (dorsal motor) nucleus of the vagus nerve, spinal tract nucleus of the trigeminal nerve] and in a further two goats (0.7%) cerebellum was not available for examination.

Of the 280 tested goats, three (1.1%) were fallen stock and the remaining 277 goats at the end of their productive life. Six (2.1%) male and 274 (97.9%) female goats were tested, which included 114 (40.7%) Saanen, 61 (21.8%) Toggenburg, 69 (24.6%) Anglo Nubian, and 36 (12.9%) British Alpine goats. Median age of the 280 goats was 48.5 months (range 18–180 months).

The prion protein genotype distribution in the tested population relevant to scrapie susceptibility is displayed in Table 1.

**Table 1:** Polymorphisms associated with classical scrapie resistance in the 280 tested goats.

**Codon**	**Polymorphisms**	**Number of goats**
142	II	146 (52.1%)
	IM^*^	112 (40.0%)
	MM^*^	22 (7.9%)
211	RR	263 (93.9%)
	RQ^*^	17 (6.1%)
222	QQ	246 (87.9%)
	QK^*^	33 (11.8%)
	KK^*^	1 (0.4%)

* *associated with partial resistance to scrapie*.

*In total, 169 (60.4%) goats had an allele or allele combinations associated with partial resistance to scrapie*.

*All goat were homozygous for H at codon 143, for N at codon 146, and for R at codon 154, which are not associated with scrapie resistance*.

Of the 34 goats with a K_222_ allele, 24 (70.6%) were Saanen, seven (20.6%) were Anglo-Nubian, two (5.9%) British Alpine, and one (2.9%) Toggenburg.

Genotyping of 54 male goats used or intended for breeding (median age 36 months, range: 7-72 months) identified four (7.4%) with QK at codon 222.

## Discussion

Based on experimental studies that indicated that the prion protein is extremely resistant to biological degradation and remains biologically active for years (19, 20), we expected that C&D on premises that housed scrapie-affected goats was unlikely to prevent re-infection of newly introduced goats. However, this was purely based on our knowledge from studies of classical scrapie in sheep, often in experimental settings, which generally involved sheep with highly susceptible genotypes. These studies have shown that the currently recommended C&D protocol did not prevent re-infection (3, 4). Epidemiological studies in Iceland suggested that the scrapie agent may have remained biologically active for years in a sheep barn despite disinfection (1). It is currently unknown whether goats are similarly at risk of re-infection through contaminated environments. This does not take into account the risk of introduction of scrapie by purchasing already infected animals. A probability model to estimate the risk of introducing scrapie from restocking goats in Great Britain suggested that mass restocking with more than 1,000 goats would almost certainly re-introduce disease (13). Records from the farms that provided the goats for restocking provided some confidence, although limited due to few goats tested from each farm by active scrapie surveillance, that the risk of introduction of scrapie through purchased goats after repopulation was low. There was no evidence that re-infection occurred, at least to a level seen when scrapie was first diagnosed in a goat on farm, which eventually resulted in whole herd cull. There are few possible scenarios: firstly, the scrapie agent was completely inactivated despite evidence in sheep studies that the agent remains biologically active for a long time, even after C&D. Secondly, the goat scrapie agent is less persistent in the environment than a sheep scrapie agent, even though the classical scrapie agent can transmit from sheep to goat (21–24) and vice-versa (25, 26) suggestive of similar strain properties. Thirdly, genetically susceptible sheep [sheep homozygous for V_136_ or Q_171_ (5)] may be more susceptible to scrapie infection than goats of similar genetic susceptibility [goats with the wild-type haplotype and not the variant associated with resistance: homozygous for I_142_, H_143_, N_146_, R_154_, R_211_ and Q_222_ (9)] based on the assumption that the scrapie agent still exists on the farm given the long persistence of TSE agents in the environment in general.

It is important to note that the negative results simply implied that scrapie prevalence was below 1% as determined by active scrapie surveillance in Great Britain where brain samples are tested by the currently used rapid test. If re-infection has in fact re-occurred to a very low level, a larger sample size would have been required. We assumed a test sensitivity of 97.4% using IHC for brain and RPLN based on the only study available at the time that used comparable tests (17), although it may be less (94.4%) as reported for a more recent study using the same methods (15). It would have been preferable to test predominantly fallen stock, which represent the population most likely to have scrapie confirmed. However, this was not possible for logistical reasons, which was taken into account when the sample size was calculated.

A total of 12.2% carried the K_222_ allele, which is associated with resistance to classical scrapie in goats (9). In the most recent survey in Great Britain, 11.5% of herds had goats with a K_222_ allele (95% confidence interval of 2.8–20.2%), and there was only one farm with two goats with this genotype (10) but the farm reported here did not participate in this survey. The presence of four male heterozygous K_222_ goats is encouraging in terms of increasing resistance to classical scrapie in goats on this farm because this polymorphism was ranked highest in terms of classical scrapie resistance based on the weight of scientific evidence and the strength of resistance (8). In addition, 6.1% had the Q_211_ haplotype (heterozygous) associated with some protection (27, 28) and almost half of the tested population had the M_142_ haplotype (homo- and heterozygous) although it provides only limited protection based on field studies in this herd at the time of herd cull (14) and other herds (29). Contrary to sheep, there has not been any legal requirement to repopulate the farm with goats that carry resistant genotypes. Consequently, it was not known at the start of the study that a large proportion of tested goats had an allele or allele combination associated with partial resistance to classical scrapie where the risk of infection may be lower (M_142_ and Q_211_ haplotype), or infection may not happen at all (K_222_ haplotype, particularly if homozygous). However, it was equally not known whether this was different to the genotype distribution of goats on farm at the time of herd cull because genotypes were determined only for a selected subset (14). Testing known susceptible goats only would have increased our confidence that scrapie has not re-occurred on the farm but at the same time the introduction of goats with genotypes associated with some protection against scrapie should have reduced the infectious pressure and thus contributed to the elimination of scrapie on this farm.

Current EU legislation requires testing of brain from fallen stock or healthy slaughtered goats for 2 years following herd cull and repopulation, which was also applied to this farm. Whilst this protocol will aid in determining the TSE status of goats that were used for restocking, it will not detect goats that have been re-infected on the premises because the survival time usually exceeds 2 years in goats. The median age of 72 confirmed cases in a subset of selected goats on this farm at the time of whole herd cull was 65 months and only one case was below 24 months, which had PrP^Sc^ only detectable in lymphoid tissue (30). Brain examination will not identify cases with peripheral PrP^Sc^ accumulation that has not yet spread to the brain, and goats from this farm were not subject to active scrapie surveillance anymore after the 2 years had passed. There was no evidence, however, that infection had since re-occurred in the new population. Goats in an earlier stage of disease should have been identified by examination of RPLN, which was the most frequently affected lymph node in studies of naturally occurring classical scrapie in goats where multiple lymph nodes were examined (14, 15). Some goats may have detectable PrP^Sc^ only in brain without evidence of peripheral distribution, which has been demonstrated in four goats on this farm at the time of herd cull (30), but these goats were relatively young with a median age of 44.5 months, so should have been detected in the present study if it occurred. In addition, this study was carried out 9–11 years after repopulation when most of the goats purchased for restocking would have been dead. All tested goats were born on the farm, including the 26 (9.3%) that were 9 years of age or older.

In summary, this study found no evidence that classical scrapie was re-introduced on this goat farm through mass restocking or inadequate C&D procedures. Further TSE surveillance of goats on this farm is desirable to confirm absence of disease, and breeding with male goats carrying the K_222_ allele should be encouraged to increase the scrapie-resistant population.

## Data Availability Statement

The data link genotype to phenotype in animal subjects only. All the raw data are presented as Supplementary Materal.

## Ethics Statement

Ethical approval by the institute's Animal Welfare Ethical Review Body was not sought because selected goats were transported dead to a regional laboratory for tissue collection between August 2017 and June 2019. Tested animals were goats over 18 months either at the end of their productive life, which were euthanized on farm by anesthetic overdose with pentobarbitone (Pentobarbital, Ayrton Saunders, Liverpool, UK, later Pentoject, Animalcare, York, UK) after sedation with Xylazine (Rompun 2%, Bayer, Reading, UK) instead of being sent for slaughter, or fallen stock. Written informed consent was obtained from the owners for the participation of their animals in this study.

## Author Contributions

The study was designed by PA, with support from BR and TK. SL, BR, and TK managed individual aspects of the study. PA and SL carried out the epidemiological investigation, LF was responsible for genotyping, and pathological examination were conducted by JS and BV. TK analyzed the data and drafted the manuscript. All authors contributed to the article and approved the submitted version.

## Conflict of Interest

The authors declare that the research was conducted in the absence of any commercial or financial relationships that could be construed as a potential conflict of interest.
